# Optimizing polypharmacy among elderly hospital patients with chronic diseases—study protocol of the cluster randomized controlled POLITE-RCT trial

**DOI:** 10.1186/s13012-014-0151-7

**Published:** 2014-10-06

**Authors:** Christin Löffler, Eva Drewelow, Susanne D Paschka, Martina Frankenstein, Julia Eger, Lisa Jatsch, Emil C Reisinger, Johannes F Hallauer, Bernd Drewelow, Karen Heidorn, Helmut Schröder, Anja Wollny, Günther Kundt, Christian Schmidt, Attila Altiner

**Affiliations:** Institute of General Practice, Rostock University Medical Center, Rostock, Germany; Hospital Pharmacy, Rostock University Medical Center, Rostock, Germany; Department of Internal Medicine II, Rostock University Medical Center, Rostock, Germany; Dietrich-Bonhoeffer Hospital, Neubrandenburg, Germany; Institute of Pharmacology, Rostock University Medical Center, Rostock, Germany; AOK Scientific Institute (WIdO), Berlin, Germany; Institute for Biostatistics and Informatics in Medicine and Ageing Research, Rostock University Medical Center, Rostock, Germany; Rostock University Medical Center, Rostock, Germany

**Keywords:** Polypharmacy, Drug utilization review, Inappropriate prescribing, Patient-centered care, Secondary care, Primary health care

## Abstract

**Background:**

Treatment of patients with multimorbidity is challenging. A rational reduction of long-term drugs can lead to decreased mortality, less acute hospital treatment, and a reduction of costs. Simplification of drug treatment schemes is also related to higher levels of patient satisfaction and adherence. The POLITE-RCT trial will test the effectiveness of an intervention aiming at reducing the number of prescribed long-term drugs among multimorbid and chronically ill patients. The intervention focuses on the interface between primary and secondary health care and includes a pharmacist-based, patient-centered medication review prior to the patient’s discharge from hospital.

**Methods:**

The POLITE-RCT trial is a cluster randomized controlled trial. Two major secondary health care providers of Mecklenburg-Western Pomerania, Germany, take part in the study. Clusters are wards of both medical centers. All wards where patients with chronic diseases and multimorbidity are regularly treated will be included. Patients aged 65+ years who take five or more prescribed long-term drugs and who are likely to spend at least 5 days in the participating hospitals will be recruited and included consecutively. Cluster-randomization takes place after a six-month baseline data collection period. Patients of the control group receive care as usual. The independent two main primary outcomes are (1) health-related quality of life (EQ-5D) and (2) the difference in the number of prescribed long-term pharmaceutical agents between intervention and control group. The secondary outcomes are appropriateness of prescribed medication (PRISCUS list, Beers Criteria, MAI), patient satisfaction (TSQM), patient empowerment (PEF-FB-9), patient autonomy (IADL), falls, re-hospitalization, and death. The points of measurement are at admission to (T0) and discharge from hospital (T1) as well as 6 and 12 months after discharge from the hospital (T2 and T3). In 42 wards, 1,626 patients will be recruited.

**Discussion:**

In case of positive evaluation, the proposed study will provide evidence for a sustainable reduction of polypharmacy by enhancing patient-centeredness and patient autonomy.

**Trial registration:**

Current Controlled Trials ISRCTN42003273

## Background

Along with demographic and epidemiologic changes, *polypharmacy* becomes a pressing challenge. More and more people suffer from chronic diseases and multimorbidity [1-3] and are treated with an increasing number of drugs. Whereas polypharmacy usually refers to the intake of five or more long-term drugs at the same time [4,5], patients with multiple chronic diseases are often treated with ten or more drugs [6-8]. In Germany, the prevalence of polypharmacy among elderly patients is about 27% and doubles to 54% when including over-the-counter medications (OTC) [9].

Treatment of patients with multimorbidity is challenging; medical guidelines tend to focus on a solitary chronic disease. In patients with several chronic diseases conflicting recommendations are likely to occur. Polypharmacy, thus, involves severe risks for patient safety; it is associated with a reduced ability to perform tasks of daily living, with an increased risk for impaired cognitive capacity, and with rising incidence of geriatric syndromes, such as delirium, falls, and urinary incontinence. In addition, patients with polypharmacy witness an increased rate of adverse drug events and drug interactions [10-12]. Among geriatric patients, 10%–15% of all hospital admissions are related to adverse drug events [13]. Adverse drug events increase both morbidity and mortality [12-15]. Previous research shows that a reasonable reduction of long-term drugs can lead to decreased mortality, less acute hospital treatment, and a reduction of costs. Simplification of drug treatment schemes is also related to higher levels of patient satisfaction and adherence [5,14,16-20].

In the past, different tools have been developed to assess and optimize medication in chronically ill patients. In 1991, Beers et al. presented a first catalogue of criteria to detect inappropriate substances among older people—the Beers Criteria for Potentially Inappropriate Medication Use in Older Adults [20,21]. Due to different “markets” and prescribing behavior, countries such as Canada, France, and Germany created their own lists of potentially inappropriate medication (PIM). In 2010, the first German PIM list was published; the PRISCUS list is based on international literature and specific characteristics of Germany. It is systematically consented by experts in the field [22,23].

Furthermore, the usage of OTCs is rising steadily. Very often, though, patients do not perceive OTCs as medication. Although nearly 30% of German adults periodically make use of herbal preparations—primarily as dietary supplements—they rarely report the usage to their doctors [24,25]. Among patients with multimorbidity, who are using a high number of drugs, these dietary supplements can cause severe drug interactions such as bleeding [26] or reduced plasma drug levels [27].

Interventions relying on a concept where pharmacists provide information to clinicians have shown effects on reducing inappropriate prescriptions [12]. Standardized prescription-feedback [28] and educational outreach visits also showed to have some effect towards the reduction of inappropriate multiple medication [12,16,17]. Studies have been conducted in different settings, however, focusing mainly on nursing homes or secondary care. Despite promising results [11,14,29,30], there is still a lack of robust evidence on the sustainability of the effects of medication reviews [31]. Whereas some trials lacked a control group [9,12], others failed to reduce polypharmacy in the long run [5,32,33]. As far as the latter are concerned, patients’ and primary care physicians’ preferences to switch back to the previous medication scheme have been identified as a major problem [34-37]. In sum, there is evidence for the efficacy of well-planned and performed interventions to reduce polypharmacy. However, evidence for effectiveness and efficiency is still lacking, especially as far as effects on patients’ quality of life are concerned [38].

### Objectives

The POLITE-RCT trial will test the effectiveness of an intervention aiming at reducing the number of prescribed long-term drugs among multimorbid and chronically ill patients aged 65+ years. The intervention focuses on the interface between primary and secondary health care and includes a pharmacist-based, patient-centered medication review prior to the patient’s discharge from hospital. This approach is innovative and particularly promising, as hospitalization of older, multimorbid and chronically ill patients is often related to an increase in the number of prescribed long-term drugs [39,40]. It will be the first trial to systematically assess the effectiveness of the described intervention which is based on both *evidence and patient preferences*. The POLITE-RCT trial is built on previous research; the POLITE pilot study proved that the intervention is feasible. Reactions of patients, pharmacists, ward physicians, and general practitioners (GPs) were positive. On average, the number of prescribed long-term pharmaceutical agents was reduced by one per patient [8,41].

## Methods

### Trial design

The POLITE-RCT trial is a cluster randomized controlled trial. Two major secondary health care providers of Mecklenburg-Western Pomerania, Germany, will take part in the study. Together, both centers with a total of four hospitals treat about 80,000 patients a year in urban and rural settings. The study will, thus, be conducted in a setting that represents the real health service situation.

### Clusters

Clusters are wards of both medical centers including medical personnel and patients cared for during the observational periods. A ward is defined as an entity with stable medical personnel. In case of responsibility of senior physicians for two or more wards, these wards will be randomized together.

#### Inclusion criteria

All wards of the participating centers where elderly patients with chronic diseases and multimorbidity are regularly treated will be included. These are, e.g., units of internal medicine, geriatrics, abdominal and vascular surgery, orthopedic surgery, and neurology.

#### Exclusion criteria

Wards currently participating in other trials or projects aiming at optimizing drug therapy will be excluded.

### Participants

#### Inclusion criteria

Patients aged 65+ years who take five or more prescribed long-term drugs that are systemically acting (topic administration excluded) and who are likely to spend at least 5 days in the participating hospitals will be recruited and included consecutively.

#### Exclusion criteria

Patients who are not able to take their medication by themselves, who are not able to give legal informed consent (e.g., due to dementia), patients with severe language difficulties, and those who suffer from deafness as well as patients taking part in another clinical trial will be excluded. Further, patients with the following diseases that usually make polypharmacotherapy unavoidable are excluded: active malignoma, acquired immunodeficiencies (HIV), and hemodialysis. Also, post-transplant patients as well as patients having a remaining life expectancy of less than 12 months will be excluded.

### Recruitment

Each week, up to 30 eligible patients will be recruited per hospital consecutively at admission. Pharmacists will inform about the study and will seek written informed consent. Recruitment will be stopped as soon as the necessary number of patients per week is reached.

### Randomization

Cluster randomization takes place after a 6-month baseline data collection period. During this period, no intervention will be performed. This allows collecting information about potential confounders and cluster imbalances. Based on that, the decision for or against stratified randomization will be made. Computer-assisted randomization will be performed by a statistician not involved in patient recruitment, data collection, and data management.

### Intervention

**Figure 1:**
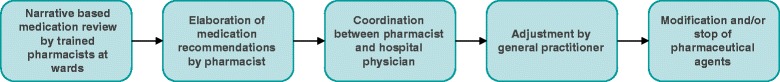
**The intervention cascade.**

During in-patient treatment of patients affected by polypharmacy, a pharmacist specially trained in communication skills performs a *narrative-based medication review*. Thus, two approaches are combined here: the face-to-face clinical “brown bag” medication review [42] and the patient-centered approach of narrative medicine [43,44]. Apart from detecting potentially inadequate medication, a major aim is to identify patient preferences and to include them—whenever possible—into a hierarchically structured list of evidence-based medication recommendations. Thus, priorities for medication modification can be based on both “objective” pharmaceutical considerations as well as on “subjective” patient preferences. The pilot study showed that this approach motivated patients to actively contribute to the reduction of medication. The *narrative-based medication review* itself takes approximately 30–45 min. The pharmacist then prepares a list of possible drugs to be stopped. Pharmacists have access to a clinical decision support system [45]. The list will be discussed with the hospital physician in charge and will be submitted for adjustment with the patient’s individual GP. The active involvement of the patient’s GP aims at bringing transparency into the decision-making and might increase chances for a sustainable optimization of medication by preventing relapse to old medication patterns (as frequently observed in other studies). See Figure 1.

### Control group

Patients attending wards randomized into the control group will not receive a medication review but receive care as usual. Medication data will be obtained from patient records. Contamination between control and intervention group will be minimized by careful and strict separation of functional units.

### Outcomes

#### Primary outcomes

The independent two main outcomes are (1) health-related quality of life (EQ-5D) [46-48] and (2) the difference in the number of prescribed long-term pharmaceutical agents between intervention and control group at T3. Since in Germany, combination drugs are frequently used, the primary outcome focuses on pharmaceutical agents rather than on number of drugs.

#### Secondary outcomes

The secondary outcomes are appropriateness of prescribed medication (PRISCUS list [22], Beers Criteria [49,50], MAI [51,52]), patient satisfaction (TSQM) [53,54], patient empowerment (PEF-FB-9) [55], patient autonomy (IADL) [56], falls (frequency and severity) [57], re-hospitalization, and death. For all patients ensured with the largest public German health insurance provider AOK, cost-effectiveness will be analyzed by the Scientific Institute of the AOK (WIdO). In addition, data on socio-demographic characteristics [58] will be collected. See Table 1.

**Table 1:** **Overview of instruments and time of measurements**

**Outcome**	**Instrument**	**Time of measurement**			
		**T0**	**T1**	**T2**	**T3**
**Primary outcomes**					
Health-related quality of life	EQ-5D	X		X	X
Difference in the number of prescribed long-term pharmaceutical agents	Number of prescribed long-term pharmaceutical agents	X	X	X	X
**Secondary outcomes**					
Appropriateness of prescribed medication	PRISCUS list, Beers Criteria	X	X	X	X
	Medication appropriateness index (MAI)	X	X	X	X
Patient satisfaction	TSQM	X		X	X
Patient empowerment	9-item shared decision-making questionnaire (PEF-FB-9)	X		X	X
Patient autonomy	Instrumental activities of daily living scale (IADL)	X		X	X
Falls	*Frequency*: number of falls in the previous 6 months	X	X	X	X
	For each fall, *severity* will be assessed:				
	*Moderate*: bruising, sprains, cuts, abrasions, or reduction in physical function for at least three days or if participant sought medical help				
	*Severe*: fractures, admission to hospital with an injury, or if stitches were required				
Re-hospitalization				X	X
Death			X	X	X
Cost-effectiveness	Difference in health care costs between both groups				X
**Additional data**					
Demographic data	Socio-demographic characteristics in epidemiological studies (SDD)	X		X	X

#### Points of measurement

Prior to the intervention, baseline data will be collected. During the following intervention period, for each patient of the intervention and control group, primary outcomes will be measured at four points in time: at admission to (T0) and discharge from hospital (T1) as well as 6 and 12 months after discharge from the hospital (T2 and T3).

### Blinding

Patients, pharmacists, physicians, study assistants, and statisticians will not be blinded. Only at T2 and T3, pharmacists will be blinded for data collection.

### Sample size

**Figure 2:**
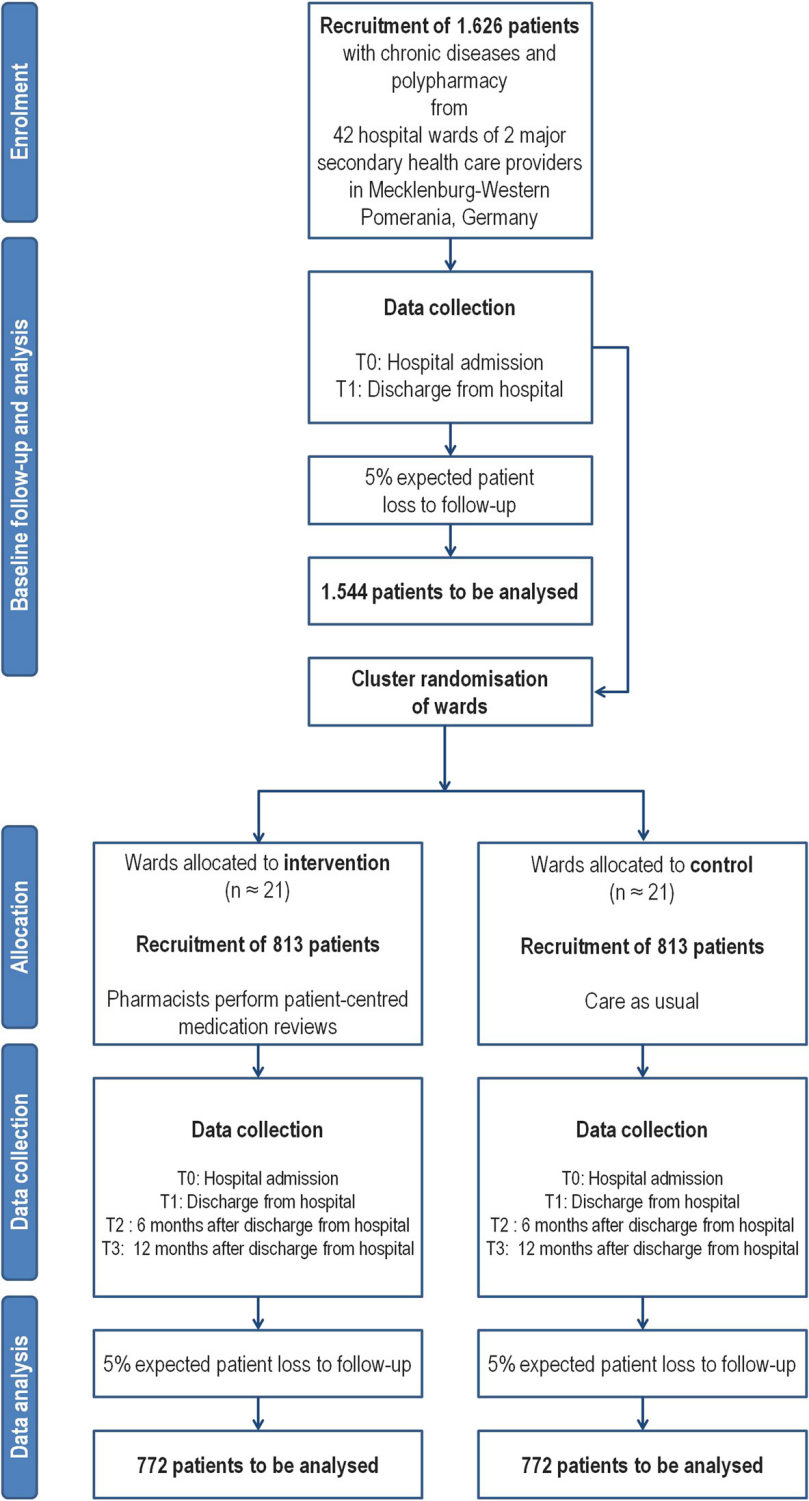
**The flowchart.**

For the two independent primary outcomes of (1) health-related quality of life (EQ-5D) and (2) the difference in prescribed long-term pharmaceutical agents between intervention and control group at 12 months after discharge (T3), the following assumptions are made: for the single summary index of the five dimensions of the EQ-5D descriptive part (using reference value sets for Germany) for the changes from T0 to T3, a mean clinical relevant difference of 0.1 with standard deviation of 0.3 shall be demonstrated between intervention and control. For the difference in prescribed long-term pharmaceutical agents between both groups at 12 months after discharge (T3), a mean difference of 0.5 (number of agents) in reduction of agents with an expected standard deviation of about 1.5 has to be proven. If an EQ-5D difference of 0.1 with standard deviation of 0.3 (or a mean difference of 0.5 in reductions of agents with an expected threefold standard deviation) is to be demonstrated with a power of 80% at a significance level of 5% two-sided, a sample size of 143 per group will be required in a randomized trial. For the cluster randomized trial, this sample size has to be multiplied by a design factor of 5.4 [59] if an intra-class correlation coefficient (ICC) of 0.1 and a cluster size of 40 patients is assumed, resulting in a sample size of 1,545 evaluable patients in 38 clusters. Assuming a dropout rate of 5%, a total sample size of 1,626 patients in 42 wards has to be recruited initially. The number of patients per cluster was conservatively defined in order to cope with possible variations that could not be taken into account at the start of the trial. Therefore, sample size estimations will be verified by results of the baseline data analysis. If necessary, adjustments of sample size estimations will be realized. See Figure 2.

### Data collection, quality, and monitoring

The personnel of the participating wards will not be involved in data collection. Instead, data collection will be performed by trained pharmacists and study assistants. Pharmacists will also perform telephone data collection at T2 and T3 and will not receive information about group affiliation of patients. To reduce selection bias, a standardized and scrupulously followed recruitment procedure will be employed, supervised by research assistants and monitored by the Clinical Trial Center North at the University Hospital Hamburg-Eppendorf (CTCN). Monitoring will be conducted according to the standard operation procedures of the Clinical Trial Center North and in compliance with the E6 ICH GCP guideline for good clinical practice. Clinical monitoring will also include review and resolution of missing or inconsistent results and source document checks (i.e., comparison of submitted study results to original reports) to assure the accuracy of the reported data.

### Statistical methods

The unit of inference will be the individual. Then, analyses will be carried out at cluster and at individual level. In a first simple approach, data in each cluster will be collapsed to construct a relevant summary measure (mean with standard deviation). This essentially removes the need to adjust for clustering effects. Of course, for variable-sized clusters, this analysis does neither take into account the intra-cluster correlation nor the cluster sizes and is less efficient than an individual-level analysis. But nevertheless, the relative simplicity of a cluster-level analysis still remains an advantage, albeit with some loss of efficiency and an inability to adjust for individual-level risk factors. In a second step, generalized estimating equations (GEE) models will be applied [60,61]. A generalized linear model takes into account within-group correlation and allows adjustment for the joint effects of cluster-level as well as individual-level factors without the requirement of parametric assumptions.

A model that takes into account the randomized clusters (wards) as random effect, with EQ-5D change or difference in prescribed long-term pharmaceutical agents between intervention and control at T3 as dependent and random group as independent variables, will be fitted to the data. In this model, baseline (T0) values of primary variables and a selection of further baseline characteristics of ward or patient will function as covariates. The intervention effects are quantified by the between-group differences of the corresponding estimates of changes from baseline from the fully adjusted model, which we assume to give the best account of the study results. In the statistical analyses, missing values will not be replaced. Methods for dealing with outliers will be defined in the statistical analysis plan (SAP) or in an addendum to the SAP. Secondary efficacy variables will be analyzed in an explorative way. Similarly, confidence intervals computed will be interpreted as interval estimates for presence or absence of effects in the study data. Intention-to-treat analyses will be performed.

### Stopping rules

Data on death (T1, T2, T3) and hospital re-admissions (T2, T3) of the included patients will be collected. If significant differences in the re-admission rate or number of deaths will be discovered, the safety board will be informed to decide whether to stop or continue the trial.

### Ethical approval

The study was approved by the Ethics Committee at the Rostock University Medical Center in June 2014 with the reference A 2014–0101.

### Study registration

The study has been registered with Current Controlled Trials Ltd. with the reference ISRCTN42003273.

### Trial status

Patient recruitment and baseline data collection started in August 2014.

## Discussion

The POLITE-RCT trial focuses on the pressing challenge of reducing inappropriate medication among elderly patients suffering from chronic diseases. The setting of the study, the German region of Mecklenburg-Western Pomerania offers the particular chance to study the effectiveness of the proposed intervention in an area particularly affected by population aging and an increasing number of people with chronic diseases and multimorbidity [62].

The intervention concept of POLITE-RCT is innovative; as hospitalization of older, multimorbid, and chronically ill patients is often related to an increase in the number of prescribed long-term drugs [39,40], the focus on the interface between secondary and primary health care is particularly promising. To our knowledge, this is the first trial that systematically assesses the effectiveness of a *narrative-based medication review* performed by trained pharmacists. Pharmaceutical recommendation will be based on both *evidence and patient preferences*.

Major methodological strengths of the trial include external monitoring as well as stratified randomization of participating wards after baseline data collection. This way, imbalances between intervention and control group will be minimized. Also, based on baseline data, sample size requirements will be adapted.

Potential sources of bias include, e.g., the risk of contamination between intervention and control wards, interviewer bias, and the risk of low inter-rater reliability. To reduce the latter, pharmacists will regularly discuss patient cases and will decide about controversial cases by consensus.

## Conclusion

The POLITE-RCT trial aims at providing robust evidence for the effectiveness of pharmacist-based narrative medication reviews used at the interface between primary and secondary health care. In case of positive evaluation, the proposed study will provide evidence for a sustainable reduction of polypharmacy by enhancing patient-centeredness and patient autonomy. Further, if the trial provides evidence for the cost-effectiveness of the intervention, policy- and other decision-makers might consider implementation into routine care.

## Abbreviations

GEE: generalized estimating equations
GP: general practitioner
ICC: intra-class correlation coefficient
OTC: over-the-counter drugs
PIM: potentially inappropriate medication
SAP: statistical analysis plan
